# Effect of Stress on Autonomic and Cardiovascular Systems in Military Population: A Systematic Review

**DOI:** 10.1155/2020/7986249

**Published:** 2020-08-11

**Authors:** Álvaro Bustamante-Sánchez, José Francisco Tornero-Aguilera, Valentín E. Fernández-Elías, Alberto J. Hormeño-Holgado, Athanasios A. Dalamitros, Vicente Javier Clemente-Suárez

**Affiliations:** ^1^Universidad Europea de Madrid, Faculty of Sport Sciences, Madrid, Spain; ^2^Studies Centre in Applied Combat (CESCA), Toledo, Spain; ^3^Laboratory of Evaluation of Human Biological Performance, School of Physical Education and Sport Sciences, Aristotle University of Thessaloniki, Thessaloniki, Greece; ^4^Grupo de Investigación en Cultura, Educación y Sociedad, Universidad de la Costa, Barranquilla, Colombia

## Abstract

Stress is regulated by the autonomous nervous system, increasing the sympathetic modulation when a threat is perceived. A multifactorial response usually leads to significant behavioural modifications and alterations on homeostasis and physical and psychological status. Moreover, stress is an emotional response that can lead to psychosocial and psychophysiological adversity. Regarding military population, military operations and combat exposure are important stressors that influence acute and chronic stress response in soldiers, affecting their performance and health. A bibliographic search was carried out between April and May 2019, focusing on recent studies (2013–2019) that analysed psychophysiological response, stress, stress regulation, heart rate, heart rate variability, and posttraumatic stress disorder in military population. Autonomic and cardiovascular chronic stress seems to be modulated by experience and previous specific training of each military unit. Physical exercise, music embedded with binaural beat technology, bidirectional sensory motor rhythm training, heart rate variability biofeedback, and transcutaneous vagal nerve stimulation are the main techniques applied to balance stress and to recover body homeostasis. Since military population are usually exposed to multiple stressors, knowing previous training and experience, together with developing techniques to balance stress, is the main practical application in this field of study to balance autonomic and cardiovascular systems.

## 1. Introduction

Stress is a multifactorial response that leads to significant behavioural modifications and alterations on homeostasis and physical and psychological status. The stress response is regulated by the autonomous nervous system, increasing the sympathetic modulation when a threat is perceived and causing increases in the physiological response (heart rate, blood pressure, breathing frequency, glucose levels, etc.). When stressors disappear, the parasympathetic modulation increases, returning the organism to the homeostasis state [1]. Stress has become a challenge for humans because of its negative physiological and psychological implications [2], which are normally related to cardiovascular diseases [3]. A chronic activation of the autonomous sympathetic nervous system could trigger acute heart diseases and hypertension [4].

In this line, posttraumatic stress disorder (PTSD) is one of the most common postdeployment chronic stress diseases [5] and usually happens after a war deployment and the highly stressful events that military personnel have to cope with. PTSD reduces the quality of life by a hyperarousal state that influences autonomic modulation. In fact, PTSD was prospectively associated with heart disease mortality among veterans free of heart disease at baseline [6]. Stress should be trained and monitored during military rehearsal: heart rate (HR) is one of the most used indexes to measure stress response, although heart rate variability (HRV) (variability of the time between R waves of the electrocardiogram or RR intervals) is more sensitive to cardiac autonomic modulation changes [7].

While there have been systematic reviews on PTSD among military and ex-military personnel, there are no systematic reviews in the acute and chronic stress response of military population and its effect on the cardiovascular and autonomic nervous system. Then, we proposed the present research with the aim to analyse the effect of acute, chronic, and regulation stress methods of soldiers in their cardiovascular and autonomic nervous system.

## 2. Materials and Methods

### 2.1. Search Strategy

The bibliographic search was carried out between April and May 2019. The electronic databases such as Web of Science, EBSCOHost, SCOPUS, and PubMed identified studies published between January 2013 and May 2019. The keywords used were as follows: “heart rate,” “heart rate variability,” “autonomic modulation,” “stress,” “military,” “combat,” “soldier,” “PTSD,” and “Armed forces.” The reference lists of included studies were checked for further relevant papers.

### 2.2. Inclusion Criteria

Inclusion criteria for the systematic review were as follows: (1) studies measuring psychophysiology response, stress, stress regulation, heart rate, heart rate variability, and PTSD; (2) studies focusing on military samples exposed to stressful situations; (3) studies published between 2013 and 2019; (4) studies published in English; and (5) the design of the studies met the level 2 (prospective cohort study and retrospective study), according to the guidance of The Journal of Bone and Joint Surgery level of evidence grading tool [8].

### 2.3. Selection Criteria

**Figure 1:**
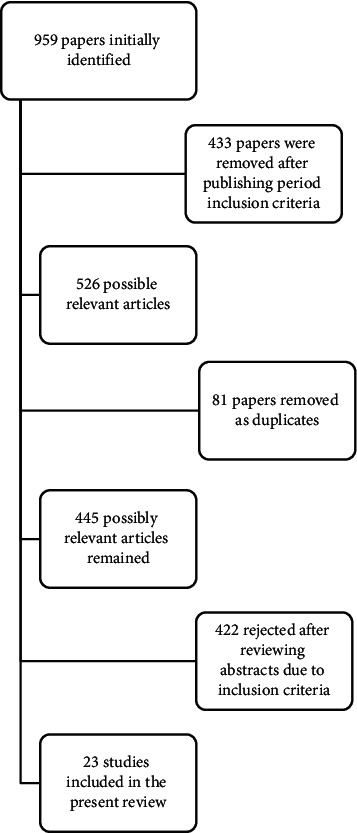
Flowchart of study search.

A total of 959 papers were retrieved from the above bibliographic searches, and 433 papers were removed after publishing period inclusion criteria. Eighty-one papers were removed as duplicates, and 422 were rejected after reviewing paper titles and abstracts. The final 23 papers were read to be considered relevant to the search criteria and appropriate for assessing our research objective (Figure 1). Reviews were used for the introduction section.

### 2.4. Exclusion Criteria

Exclusion criteria were as follows: (1) reviews, PhD dissertations, conference proceedings, abstracts, unpublished studies and books, study cases, and papers not in English.

## 3. Results and Discussion

The general definition of “stress” focuses on acute intense situations as in the fight-or-flight response presented in military population during combat and combat simulation. However, the brain, as the central organ managing stress, since it perceives what is threatening, as well as the behavioural and physiological responses to the stressor, leads to adaptations (i.e., “allostasis”) but also contributes to pathophysiology (“allostatic load/overload”) when overused and dysregulated [9]. Thus, when the stressor keeps affecting the psychophysiological response over time, the stress response becomes chronic.

### 3.1. Stress Response in the Military Population

Regarding military population, military operations and combat training simulation exposure are important stressors that influence chronic stress response in soldiers, affecting their performance and physical integrity and health.

Paratroopers are a military corps that suffers the stress of parachute jumping and combat. Regarding the parachute jump, two studies have analysed the effects of chronic stress response comparing experienced and novice paratrooper. In the first study, the psychophysiological response and specific motor skills of 17 novel and 23 expert warfighters before and after a tactical combat parachute jump were analysed [10]. They found a 1.7% greater increase in HR, 38.7% in salivary cortisol, 52.5% in creatine kinase, and 188.9% in lactate in novel paratrooper compared to expert ones. However, in any case, fine motor skills and muscle performance were affected. In the second study, conducted by the same research group, the psychophysiological response of 11 sports parachute jumps, 8 manual tactical parachute jumps, and 4 tandem pilot and 4 tandem passengers parachute jumps was compared. In this case, only the 4 tandem passengers were novice with the rest of the paratroopers having more than 30 jumps experience. It was found that novice parachute jumpers presented a higher psychophysiological stress response before and after the parachute jumps than the experienced jumpers. Also, they presented a large anticipatory anxiety response before the jump, but it was decreased afterwards [11]. In the case of parachute jumps, it seems that experience modulates chronic stress response, reducing the psychophysiological and cardiovascular anticipatory and during the task responses, although it does not seem to affect specific performance.

Infantry units and other ground forces usually use simulated combat as training for real combat situations. Within these methods, new operation theatres, such as close-quarter combat, underground operations, or urban combat, examples of asymmetrical combat, are defined as highly stressful combat situations in which there are a large number of uncontrolled threats (urban areas, the presence of civilians in the battlefield, and unstructured and undefined battlefield [12]). Chronical exposure to this kind of stressors can drive soldiers to PTSD or other diseases. The effect of chronic stress on soldier's performance has been studied compared with nonelite soldier with less or no previous exposure to different combat situations. The effect of combat stress on psychophysiological responses and performance of elite and nonelite infantry soldiers was analysed during a tactical combat developed in urban area [13]. They found the elite soldiers presented a significantly higher lactate concentration after combat than nonelite soldiers (3.8 ± 1.5 vs. 6.6 ± 1.3 mmol/L). Nonelite soldiers had a higher heart rate before and after the simulation than elite soldiers (82.9 ± 12.3 vs. 64.4 ± 11 bpm, pre nonelite and elite, respectively; 93.0 ± 12.8 vs. 88 ± 13.8 bpm post nonelite and elite, respectively). Also, elite soldiers presented higher muscular strength than nonelite before and after the combat simulation. Nonetheless, cortical arousal was not significantly modified in any group. In a subsequent study, the effect of combat stress on psychophysiological response attention and memory of experienced and novel infantry soldiers during urban combat was analysed [14]. They found a significant increase in the low-frequency domain and a significant decrease in the high-frequency domain of the heart rate variability of experienced and highly trained soldiers. Also, they found that experienced had significantly higher values in blood lactate, blood glucose, blood oxygen saturation, rated perceived exertion, heart rate, and cognitive and somatic anxiety. However, the postmission questionnaire showed that experienced soldiers presented a higher negative effect on memory probably due to the highest psychophysiological activation, which seems to be related to chronic stress exposure.

This higher sympathetic activation before and during the combat was also found during urban combat simulation when comparing light infantry soldiers [15], more experienced in asymmetrical combat, with heavy infantry soldiers, less accustomed to close-quarter combat. Moreover, light infantry showed lower metabolic, cardiovascular, and anxiogenic responses before and after the combat simulation than heavy infantry. Nevertheless, fine motor skill measured by time of ammunition of a pistol magazine was similar in both groups before (39.25 ± 7.62 and 35.38 ± 8.19 s for light and heavy infantry, respectively) and after combat (31.58 ± 5.12 and 28.79 ± 4.77 s for light and heavy infantry, respectively). However, when comparing elite vs. nonelite soldiers during close-quarter combat [15], these same researchers found that the higher metabolic, cardiovascular, and anxiogenic responses viewed in elite soldiers supposed a significant loss in the fine motor skill after the combat manoeuvre (−8.34% vs. −11.23% of change in gun reloading time of elite group and novel group (*p* < 0.05)). These differences between studies may lean on the greater training and experiences of heavy infantry compared to nonelite soldiers despite the differences in the task specificity. Finally, a study comparing experienced soldiers and civilians psychophysiological and memory responses during an underground combat simulation, with interference of night-vision systems use or not, and previous fire situation or not, showed that, before the simulation, soldiers' sympathetic modulation was greater than civilians and that all groups increased their psychophysiological response and deteriorated their memory and time consciousness after simulation [16]. However, the civilian control group presented the highest number of incorrect answers on the postmission memory recall questionnaire (73% vs. 47.5, 65 and 57.8% for the soldiers' groups). Thus, it seems that experience disposes soldiers towards better self-confidence and readiness despite the effect on chronic stress that increased the anticipatory higher metabolic, cardiovascular, and anxiogenic response previous to combat. This activation seems to lead to a better fine motor skill. Nevertheless, this higher level of self-confidence and readiness does not translate into better physical performance when compared with nonelite soldiers or civilians.

Several studies have analysed stress response, mainly through questionnaires, heart rate (HR), or heart rate variability (HRV) in military population. In all cases, HR and/or HRV measures were included to evaluate stress. Mostly, male population was used, although in three cases [14, 16, 17] female soldiers were also examined. Only one study included a control group in the experimental design [14]. Standard methods using specific software for HRV analysis were implemented in almost all studies. In the majority of the selected studies, stress responses were investigated during ground operations, except one study that focused on air force soldiers in which HR and perceived stress augmented after a combat jet manoeuvre in professional pilots [18] and four studies in which participants were engaged in parachute jumps [10, 11, 19, 20].

Generally, researchers were interested in analysing acute stress responses in terms of different levels of expertise [10, 11, 13, 16]. In one of these studies, HR augmented after a parachute jump in both novice and experienced warfighters, although the psychophysiological response was higher in the less experienced group, which had less self-confidence, and more somatic anxiety with higher blood lactate levels [10]. There were no differences when examining parachute jump modalities, among sport parachute jump, manual tactical parachute jump, tandem pilots, and tandem passengers, but they were when considering the experience of the jumpers. The less experienced jumpers had a higher psychophysiological response and higher values of anxiety before the jump [11]. Underground operations also produced a stress response (blood lactate, blood oxygen saturation, rated perceived exertion, heart rate, cognitive and somatic anxiety, and sympathetic modulation) in soldiers with different experimental conditions of fire night vision, with no differences between the groups with different equipment, but a negative effect on memory modulated by previous experience [16]. Combat stress was also addressed in elite and nonelite soldiers, with higher values of heart rate for nonelite in pre- and postmeasures, but similar cortical arousal values [13].

Researchers also focused on studying units with different training backgrounds [14, 15, 21]. In one of these studies, hypoxia induced different cognitive performances for different tasks, according to the job profile (transport pilots, fighter pilots, helicopter pilots, and transport aircrew). Although HR increases due to a lower blood oxygen saturation, hypoxia did not affect HR and HRV differently when the groups were compared [21]. Light infantry and heavy infantry units have been assessed in combat simulation: light infantry training background involved a different stress response with less anxiety and lower metabolic and cardiovascular stress, both before and after the combat manoeuvre [15]. Highly trained soldiers (5.9 ± 0.8 years) had a higher stress response than lower trained soldiers (3.9 ± 3 years), with more cognitive and memory impairment and a higher physiological activation [14].

In the cases where a single task was analysed [10, 11, 13, 15, 16, 19, 20, 22, 23], data collection was performed during 2 occasions of the selected task, i.e., before and during/after. One of the studies monitored stress during four consecutive days, finding higher stress after a simulated air accident manoeuvre, with questionnaire and HRV analysis [17]. Regarding the acute stress responses in the parameters measured, a reduction in high-frequency HRV with a concurrent increase in low-frequency HRV was identified [14, 15, 19, 20, 22, 23]. Moreover, experienced soldiers presented lower HR values during a stressful situation [10, 13, 16] compared to their less experienced counterparts, or no significant changes after a parachute jump [11]. In contrast, the single study that included a control group noted a significant decrease in all the variables evaluated during HRV analysis [14].

Table 1 shows a summary of the articles related to stress response in the military population that met the level 2 (prospective cohort study and retrospective study), according to the guidance of The Journal of Bone and Joint Surgery level of evidence grading tool [8]:

### 3.2. Regulation of Stress Methods

Stress as an emotional response with adaptive function can lead to psychosocial and psychophysiological adversity. It is directly linked with a dysregulation of the autonomic nervous system, which leads to a disruption of body homeostasis and may lead to pathological conditions and syndromes due to either acute or chronic psychophysiological changes. Since no one is stranger to stress and its effects, recently, authors have tried to identify new mechanisms and intervention programs in order to improve stress management.

Physical exercise, when correctly periodized and regularly practiced, can modify the cardiac autonomic balance by increasing the parasympathetic activity and decreasing the sympathetic activity, promoting the autonomic nervous system function to meet the demands of the cardiovascular system, thus, HRV. Authors found just after 12 weeks significant increases in the vagal tone, thus increases in the parasympathetic activity, consequence of high-intensity exercise programs intervention, and however, greater significant increases and peak vagal tone was obtained at 20-week intervention program [24]. In comparison with other modalities of physical exercise, those in which the workload is interval and intense are those which have greater benefit in the autonomic nervous system and vagal tone as in sport modalities like Judo [25].

Despite physical exercise, the use of technology for stress treatment and management is largely extended. The use of music embedded with binaural beat technology (BBT) with a focus on the theta brainwave frequency was studied, as an effective, noninvasive tool with great potential on stress reduction and management. Subjects, under the intervention program (PTSD diagnosed soldiers), presented increased parasympathetic activation and decreased sympathetic response, showing greater self-reported relaxation, after using this technology [26]. In another research study, a noninvasive acoustic stimulation in PTSD patients showed improvements in SDNN, HF, LF, and systolic and diastolic blood pressure and reduction in C-reactive protein (CRP), angiotensin II to angiotensin 1–7 ratio, and interleukin-10. It was based on real-time translation of dominant brain frequencies into audible tones of variable pitch and timing to support the autocalibration of neural oscillations. PTSD symptomatology, insomnia, depressive mood, and anxiety were reduced after 6-month intervention program [27]. The increasing and growing interest in the application of psychophysiological signals and biofeedback is clear.

Bidirectional sensory motor rhythm training (SMR) and heart rate variability biofeedback were used, which allowed subjects after 21 training sessions, to control their SMR frequency bidirectionally, showing significant improvements in stress management [28]. However, no significant effects were seen in sleep quality improvement, possibly explained since bidirectional training does not result in the same neuroplastic changes seen with unidirectional training, due to the constant changing contingencies (i.e., up vs. down required) [28]. However, other simpler interventions have proven efficiency on the reduction of global PTSD symptoms with a high degree of adherence to the program, like the self-controlled HRV biofeedback 4-week intervention program [29], or relaxation and personal perception intervention programs, such as mind fullness, breathing techniques, the combination of both, and sitting quietly form [30]. In addition, the PRESIST program, also noninvasive and based on a sum of the aforementioned research studies [31] and consisting of (i) educational materials on combat and operation stress control, (ii) copying skills training involving focused and relaxation breathing exercises with biofeedback, and (iii) exposure to video multimedia stress environment to practice knowledge and skills learned in the aforementioned steps (i) and (ii), showed a protective effect on PTSD development and stress management, thus being a potential preventive strategy tool.

Finally, other methods such as transcutaneous vagal nerve stimulation (tVNS) have presented significant positive effects on systems underlying emotional dysregulation, thus improving global stress and PTSD symptoms [32], where high-frequency heart rate variability during a tilt-table procedure derived from an electrocardiogram, and skin conductance changes in response to acoustic startle while viewing emotional images, resulted in improvements in the vagal tone and moderation of autonomic response, consistent with modulation of autonomic state and response to stress. However, the use of this technology may not be accessible for everyone, thus simpler approaches which have shown significant improvement in stress management and reduction as well as PTSD global symptoms [32].

Table 2 shows a summary of the articles related to methods to regulate the stress that met the level 2 (prospective cohort study and retrospective study), according to the guidance of The Journal of Bone and Joint Surgery level of evidence grading tool [8]:

## 4. Conclusions

Soldiers' autonomic and cardiovascular chronic stress seems to be modulated by experience and previous specific training, but its magnitude can be dependent on the task performed (e.g., fine motor skills or cardiometabolic performance) and on the type of units and the specific stressors that are exposed (e.g., paratroopers or infantry).

Physical exercise, music embedded with binaural beat technology, bidirectional sensory motor rhythm training, heart rate variability biofeedback, and transcutaneous vagal nerve stimulation are the main techniques applied to modify the cardiac autonomic balance by increasing the parasympathetic activity, thus balancing stress and reaching body homeostasis with basal levels of stress.

This information could help to improve the training guidelines to follow in different military populations to adapt better to the contexts of stress that affect both autonomic and cardiovascular systems that the soldiers must face during their job. Moreover, the information of this study could help to recognize the different strategies to reduce the posttraumatic stress that veteran soldiers have to face once they have finished their professional life.

## Figures and Tables

**Table 1:** Summary of articles about stress response in military population.

Authors and year	Study title	Participants	Aim of study/assessment	Main outcomes
Clemente-Suárez et al. (2016) [10]	Experience modulates the psychophysiological response of airborne warfighters during a tactical combat parachute jump	40 male warfighters divided in two groups: novels (*n* = 17) and experts (*n* = 23)	To analyse the effect of experience in the psychophysiological response and fine motor skills of novel and expert parachute warfighters during a combat	Experience influences the psychophysiological response. Novel paratroopers were more affected than experts

Clemente-Suárez et al. (2017) [11]	Psychophysiological response in parachute jumps, the effect of experience and type of jump	27 male airborne brigade in parachute jump (*n*: 11; 41.0 ± 9.7 years), manual tactical parachute jump (*n*: 8; 33.1 ± 5.3 years), tandem pilots (*n*: 4; 35.5 ± 3.0 years), and tandem passengers (*n*: 4; 28.5 ± 5.4 years)	To analyse the effect of experience and jump on the psychophysiological response	Novice parachute jumpers had higher values of stress than the experienced jumpers, and a large anticipatory anxiety response before the jump

Clemente-Suárez and Robles-Pérez (2013) [12]	Mechanical, physical, and physiological analysis of symmetrical and asymmetrical combat	20 soldiers from the Spanish Army and Spanish Forces and Security Corps (34.5 ± 4.2 years; 176.4 ± 8.4 cm; 74.6 ± 8.7 kg; 63.3 ± 8.0 kg muscular mass; 7.6 ± 3.2 kg fat mass)	To analyse physical, mechanical, and physiological parameters during symmetrical and asymmetrical combat simulations	Asymmetrical combat showed higher maximum speed, number of sprints, sprint distance, and average heart rate. Symmetric combat presented a higher number of impacts and training load

Tornero-Aguilera et al. (2017) [13]	Effect of combat stress in the psychophysiological response of elite and non-elite soldiers	40 warfighters divided in two groups: elite (*n*: 20; 28.5 ± 6.38 years) and nonelite (*n*:20; 31.94 ± 6.24 years)	To analyse the effect of combat stress in the psychophysiological responses of elite and nonelite soldiers	Elite soldiers had higher muscular strength than nonelite in all tests (before and after the combat simulation), while cortical arousal was not modified significantly in both groups

Tornero-Aguilera et al. (2018) [14]	Use of psychophysiological portable devices to analyse stress response in different experienced soldiers	49 soldiers of Spanish Army (19 men and 1 woman; 34.5 ± 4.2 years; 176.4 ± 8.4 cm; 74.6 ± 8.7 kg; 63.3 ± 8.0 kg muscular mass; 7.6 ± 3.2 kg fat mass)	To analyse the effect of experience and training in psychophysiological response and attention and memory of soldiers in combat	The most experienced soldiers presented higher physiological activation as well as cognitive and memory impairment than lower experienced soldiers, and memory function was modulated by the type of external stimulus

Sánchez-Molina et al. (2018) [15]	Assessment of psychophysiological response and specific fine motor skills in combat units	31 male soldiers of the Spanish Army, 19 nonexperienced soldiers (30.2 ± 5.25 years, 9.95 ± 5.17 years of experience) and 12 experienced soldiers (34.5 ± 4.85 years, 14.58 ± 4.87 years of experience)	To analyse the psychophysiological response and specific motor skills in an urban combat simulation with two infantry units with different previous training and experience	A combat simulation changed the psychophysiological basal state and unbalanced the sympathetic-vagal interaction, but motor skills were not affected after the combat

Tornero-Aguilera and Clemente-Suárez (2018) [16]	Effect of experience, equipment and fire actions in psychophysiological response and memory of soldiers in actual underground operations	Fifty-four professional soldiers of the Spanish Army (mean age 30.60 ± 4.6 years; 8.85 ± 4.1 years of experience) and 16 were civilians (mean age 26 ± 3 years)	To analyse the effect of underground operations on the psychophysiological and memory response of soldiers depending on the previous experience and the use of nocturne vision systems	The underground operation produced a significant increase in blood lactate, blood oxygen saturation, rated perceived exertion, heart rate, cognitive and somatic anxiety, and sympathetic modulation in all groups
Hormeño-Holgado et al. (2019) [17]	Psychophysiological response of air mobile protection teams in an air accident manoeuvre	12 male and 1 female soldiers from an air security force unit of the Spanish Air Force (32.4 ± 8.0 years; 7.2 ± 4.8 years of experience)	To study the psychophysiological response of an air security force in a simulated air accident in a hostile area and its subterfuge to a safe area	An air accident manoeuvre of three nights and four days caused a higher sympathetic nervous system modulation and increased stress, muscle strength, and dehydration

Hormeño-Holgado and Clemente-Suárez (2019) [18]	Effect of different combat jet manoeuvres in the psychophysiological response of professional pilots	29 fighter pilots of the Spanish Air Forces (28.3 ± 7.4 years)	To analyse the effect of air combat manoeuvres (defence and attack) on the psychophysiological response of air combat fighter pilots	The defensive manoeuvre produced a significant decrease in forced vital capacity and an increase in heart rate, stress, and exertion in both manoeuvres

Clemente-Suárez et al. (2017) [19]	Psychophysiological response and fine motor skills in high-altitude parachute jumps	16 veteran male soldiers of the Spanish Army with more than 200 parachute jumps experience 8 high-altitude low-opening (32.6 ± 7.7 years) and 8 high-altitude high-opening (30.3 ± 5.6 years)	To analyse the psychophysiological response and specific fine motor skill of an experienced jumper in high-altitude low-opening and high-altitude high-opening parachute jumps	High-altitude low-opening and high-altitude high-opening jumps produced a significant increase in CK, lactate, and RPE and a decrease in glucose. High-altitude high-opening decreased cortical arousal and presented a higher sympathetic modulation and a higher HR during the jump than high-altitude low-opening

Clemente-Suárez et al. (2016) [20]	Psychophysiological response in an automatic parachute jump	We analysed 38 male sport active soldiers of Spanish Army (25.6 ± 5.9 years; 172.3 ± 4.7 cm; 70.3 ± 4.9 kg; 23.8 ± 0.5 BMI) with an average of 44.7 ± 82.1 civil and military parachute jumps	To analyse modifications in blood oxygen saturation, heart rate, cortisol, glucose, lactate, creatine kinase, muscle strength, cortical arousal, autonomic modulation, and anxiety before and after an automatic open parachute jump	An automatic parachute jump increased physiological and cortical response and decreased somatic anxiety of participants

Bustamante-Sánchez et al. (2019) [21]	Psychophysiological response of different aircrew in normobaric hypoxia training	22 male pilots (10 helicopter pilots, 7 transport aircrew, 3 transport pilots, and 3 fighter pilots) from the Spanish Air Forces	To study the effect of hypoxia training in cortical arousal, autonomic modulation, muscle strength, and cognitive function	Hypoxia produced an increase in perceived stress and effort, a higher heart rate, and a decreased function of breathing muscles. Working memory and pattern recognition were impaired after hypoxia exposition. Aircrew groups performed differently in cognitive tests, suggesting differences in their previous training

Delgado-Morerno et al. (2017) [22]	Combat stress decreases memory of warfighters in action	Twenty male soldiers from the Spanish Army (35.4 ± 6.2 years; 179.9 ± 7.0 cm; 82.3.8 ± 10.5 kg; BMI: 25.7 ± 2.6; 14.6 ± 6.4 years of experience)	To analyse the effect of combat stress in the psychophysiological response and attention and memory of warfighters in a simulated combat situation	Combat stress increased the psychophysiological response and caused a selective decrease of memory, depending on the dangerous or harmless nature of the stimulus

Gamble et al. (2018) [23]	Different profiles of decision making and physiology under varying levels of stress in trained military personnel	26 male active duty US Army Infantrymen (age = 30.73 ± 7.71 years)	To examine the relationship between decision making and physiology under varying levels of stress in trained military personnel	Participants performed worse in the high-stress condition, and heart rate variability measurements could help to measure the adaptive response when danger is imminent

**Table 2:** Summary of articles about stress regulation.

Authors and year	Study title	Sex/participants/age	Aim of study/assessment	Main outcomes
Grant et al. (2018) [24]	The difference between exercise-induced autonomic and Fitness changes measured after 12 and 20 weeks of medium-to-high intensity military training	154 healthy recruits (male = 89, female = 65, age = 20.91 ± 1.29 with a body mass index of 22.85 ± 2.78 kg/m^2^)	To compare the physical fitness, based on VO2max and exercise-induced cardiac autonomic changes, measured by heart rate variability of 12 weeks with 20 weeks of training in the South African National Defence Force	Cardiorespiratory fitness (VO2max) did not increase during the 12- to 20-week period although heart rate and sympathetic cardiac control decreased with a simultaneous increase in vagal cardiac control

Campos et al. (2018) [25]	Influence of autonomic control on the specific intermittent performance of judo athletes	Sixteen judo athletes of both sexes (12 men and 4 women, age of 19.6 ± 2.9 years, body mass of 67.9 ± 12.1 kg)	To verify the correlation between heart rate variability at rest with performance in the special judo fitness test	The rates of vagal tone in the time domain of resting heart rate variability correlated positively with the performance of judo athletes (number of throws)

Gantt et al. (2017) [26]	The effect of binaural beat technology on the cardiovascular stress response in military service members with postdeployment stress	74 military service members with a complaint of continued stress following a deployment	To assess the efficacy of embedded theta brainwave frequency in music using binaural beat technology compared to music alone on the cardiovascular stress response in military service members with postdeployment stress	Participants who used music with embedded binaural beat technology displayed a decrease in sympathetic responses and an increase in parasympathetic responses, while participants who used music alone had the opposite effect

Tegeler et al. (2017) [27]	Successful use of closed-loop allostatic neurotechnology for post-traumatic stress symptoms in military personnel: self-reported and autonomic improvements	Eighteen service members or recent veterans (15 active duty and 3 veterans, most from special operations, 1 female, age = 40.9 ± 6.9 years) and symptoms of posttraumatic stress disorder from 1 to 25 years	To document changes in self-reported symptoms, autonomic, and functional measures after use of a closed-loop acoustic stimulation neurotechnology	There were significant improvements in multiple measures of heart rate variability in both time and frequency domains

Binsch et al. (2017) [28]	No effects of successful bidirectional SMR feedback training on objective and subjective sleep in healthy subjects	62 participants, all military working at the Dutch Ministry of Defence	To analyse to what extent participants could gain voluntary control over sleep-related parameters and secondarily to assess possible influences of this training on sleep metrics	After the training, the heart rate variability values improved, but no effects were found on sleep spindles, actigraphy, sleep diaries, and self-reported sleep quality

Wahbeh et al. (2016) [30]	Mechanistic pathways of mindfulness meditation in combat veterans with posttraumatic stress disorder	102 combat veterans with posttraumatic stress disorder	To evaluate the effect of two common components of meditation (mindfulness and slow breathing) on potential mechanistic pathways	Meditation helped to improve posttraumatic stress disorder and related symptoms, although there were no different effects between groups

Hourani et al. (2016) [31]	Toward preventing post-traumatic stress disorder: development and testing of a pilot predeployment stress inoculation training program	351 active duty male Marines scheduled for imminent deployment for combat operations	To design, develop, and evaluate a predeployment stress inoculation training preventive intervention to enable deploying personnel to cope better with combat-related stressors and mitigate the negative effects of trauma exposure	The predeployment stress inoculation training protected against post-traumatic stress disorders among Marines without baseline mental health problems. This strategy could be used as a potential preventive strategy in the military personnel
Lamb et al. (2017) [32]	Non-invasive vagal nerve stimulation effects on hyperarousal and autonomic state in patients with posttraumatic stress disorder and history of mild traumatic brain injury: preliminary evidence	Participants diagnosed with posttraumatic stress disorder (*n* = 12, 30.4 ± 5.4 years) and healthy combat controls (*n* = 10, age = 29.7 ± 7.0 years)	To evaluate noninvasive vagal nerve stimulation on hyperarousal and autonomic state in patients with posttraumatic stress disorder	The stimulation improved the vagal tone and moderated the autonomic response to startle and stress in this population
